# Dissecting Aneurysms of Bilateral Anterior Cerebral Artery Complicated by Subarachnoid Hemorrhage After Cerebral Infarction: A Case Report

**DOI:** 10.4137/ccrep.s833

**Published:** 2008-07-02

**Authors:** Akihiro Kurosu, Shizuo Hatashita, Hideo Ueno

**Affiliations:** Department of Neurosurgery, Juntendo University Urayasu Hospital, 2-1-1, Tomioka, Urayasu, Chiba 279-0021, Japan.

## Abstract

**Introduction:**

Intracranial dissecting aneurysms have been increased due to recent advancements in diagnostic imaging. However there have been little article with subarachnoid hemorrhage and cerebral infarction occurring almost at the same time. We performed the surgical treatment and obtained good result.

**Case presentation:**

A 47-year-old male presented to our hospital with chief complaints of sudden headache and mild paralysis of the left lower extremity. Brain imaging at admission revealed cerebral infarction in the right frontal lobe and subarachnoid hemorrhage in the frontal convexy and anterior interhemispheric fissure. The left and right internal carotid angiography showed a bulging cerebral aneurysm at the left A1–A2 junction and stenosis and arterial dissections in the peripheral of the bilateral anterior cerebral artery. Wrapping was performed for the dissecting aneurysm of the left anterior cerebral artery. For the right anterior cerebral artery, trapping was performed at the A2 segment without vascular anastomosis. The patient’s postoperative course was uneventful.

**Conclusion:**

A consensus has not been reached on the treatment for intracranial dissecting aneurysms. Proximal trapping without vascular reconstruction was performed for the right anterior cerebral artery without vascular anastomosis to prevent rebleeding. However no symptoms of neurological deficiency were observed. Proximal trapping of dissecting aneurysm seems to be a good option when patient’s functional and life prognosis are taken into account in case that vascular reconstruction will be anticipated difficulty.

## Introduction

Reported cases in which intracranial dissecting aneurysm (DA) are detected have been increasing with non-invasive examination due to recent advancements in diagnostic imaging. In this report, we describe a patient with DAs in the bilateral anterior cerebral arteries and in whom hemorrhage and infarction occurred almost at the same time. We were able to make a definitive diagnosis by 3 Dimension—Computed Tomography (3D-CT) angiography in which dissecting intima was observed in the aneurysm. We obtained good results in this case by surgical treatment. We report the clinical conditions and treatment provided to the case of DAs in the bilateral anterior cerebral arteries.

## Case Presentation

**Figure 1:**
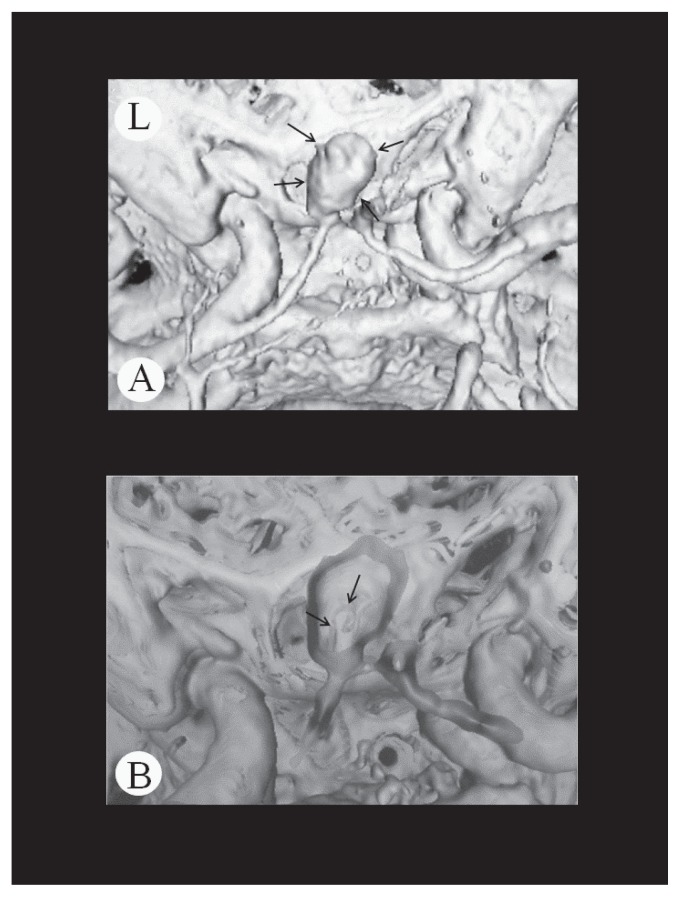
3 Dimension-Computed Tomography angiography showed saccular aneurysmal dilatation in the left A1–A2 junction (arrow), and bilateral A2 segment were ill-defined (**A**). In the cross section of the aneurysm, dissecting intima was observed (arrow) (**B**).

**Figure 2:**
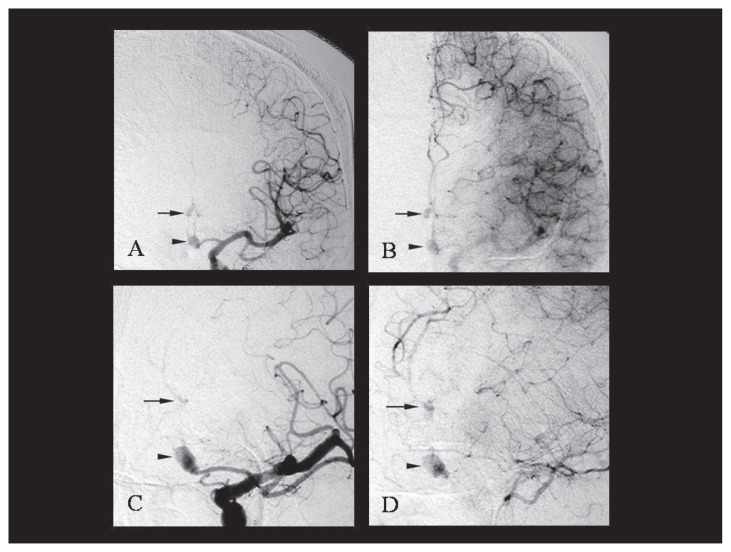
Left internal carotid angiography demonstrated saccular aneurysmal dilatation in the left A1–A2 junction (arrowhead) (**A** and **C**). At its periphery, a small saccular bulge was observed unclearly (arrow). Late arterial phase showed retrograde filling of peripheral portion of left ACA and a small saccular bulge in the peripheral portion was detected better (**B** and **D**).

**Figure 3:**
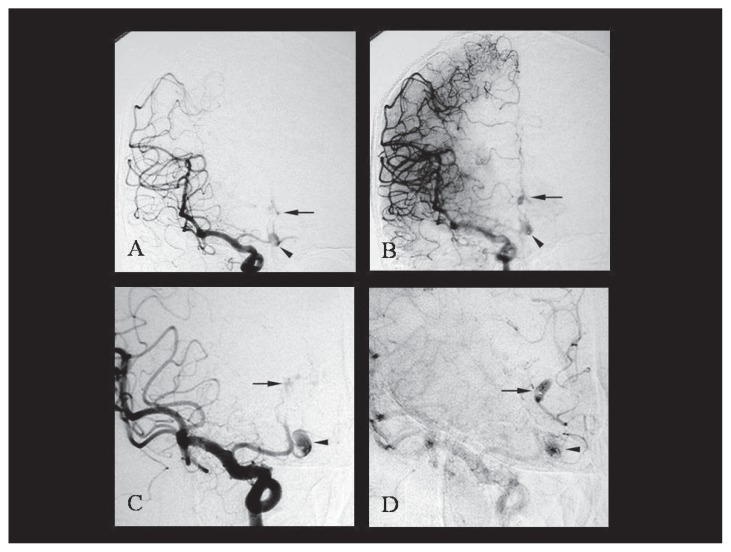
Right internal carotid angiography demonstrated saccular aneurysmal dilatation in the left A1–A2 junction (arrowhead) (**A** and **C**). The pericallosal artery were occluded and late arterial to capillary phase revealed retrograde filling of peripheral portion of left ACA and small saccular bulge (arrow) (**B** and **D**).

The patient was a 47-year-old male with non-contributory past medical history. He noticed weakness in the left lower extremity upon rising from bed in the morning, but the symptom gradually resolved. On the night of the same day, he suddenly developed headache and mild paralysis of the left lower extremity and presented to our hospital. On admission, the patient was alert and had mild paralysis of the left lower extremity. His blood pressure was 138/78 mm Hg. There were no abnormalities in the blood biochemistry tests taken upon arrival. The head CT and MRI showed cerebral infarctions in the right frontal lobe and subarachnoid hemorrhage in the frontal convexity and anterior interhemispheric fissure. In MRA, DAs were observed in the bilateral anterior cerebral arteries. In the 3D-CT angiography, dissecting intima was observed in the area with saccular enlargement (Fig. 1). The patient was diagnosed with a combination of cerebral infarction and subarachnoid hemorrhage caused by the rupture of the DAs in the bilateral anterior cerebral arteries. In the left internal carotid angiography, saccular cerebral aneurysm was observed in the left A1–A2 junction (Fig. 2). In addition, stenosis was seen in the left A2 segment and opaque vascular bulging was seen in its peripheral in the late arterial phase. Right internal carotid angiography showed cerebral aneurysm at the left A1–A2 junction (Fig. 3). The right A2 segment was occluded and its peripheral vessels showed retrograde flow during the venous phase. The treatment plan included surgery to prevent recurrent hemorrhage and to reinforce the walls of the DAs in the anterior cerebral arteries. Surgery was performed on the fifteenth day after admission when the patient’s neurological symptoms stabilized. First, anterior interhemispheric approach in a supine position was used to confirm the presence of aneurysms in the bilateral areas from A2 to A3 segments. The arterial wall was dark purple, and the vascular diameters were irregular with bulging in one area. Wrapping was performed from A2 to A3 segments for the left anterior cerebral artery. The right peripheral vessels had weak walls and vascular anastomosis was difficult. Therefore, trapping was performed at the A2 segment. In the postoperative course, the patient had no apparent neurological deterioration. One month after the surgery his symptoms had resolved completely, and he could return to work.

## Discussion

Reported cases of intracranial DA are increasing due to recent advances in non-invasive neuroimaging equipment. Generally DA in the vertebral artery circulation often produces subarachnoid hemorrhage.[1] In contrast, DA in the internal carotid artery circulation often leads to infarction.[2] When comparing infarction cases to hemorrhage cases, conservative therapy has been relatively favorable for cerebral infarction patients in some reports,[3] whereas some papers said that aggressive treatment is required for patients with hemorrhagic accident.[4] Metso et al. reported that aneurysmal artery dissection is characterized by SAH and poor prognosis, therefore in such cases, more vigorous therapy is indicated.[5]

There has not been a consensus on the treatment method for ruptured DA in internal carotid artery circulation. For cases that underwent surgery for DA at the A1 segment, trapping was performed in most cases when communication existed with the anterior communicating artery and was less likely to cause ischemic neurological deterioration.[3, 6] Most of the cases demonstrating DA in the periphery of the A2 segment were treated conservatively.[2, 6, 7] There were only six articles in the literature in which DA occurred in the periphery of the A2 segment and was treated surgically, three cases underwent trapping with revascularization,[8] and two underwent trapping without revascularization.[9] Another one case underwent embolization of DA with endovascular surgery.[4] The postoperative course was uneventful in all cases. As one reason for this is that the patients had already presented some ischemic symptoms so that new ischemic symptoms might be difficult to recognize even if such symptoms occurred. Sturzenegger et al. reported that complete or partial recanalization occurred in half of the initially occluded arteries, and complete recanalization in three-fourths of initially stenosed intracranial arteries.[10] Therefore, another reason is considered that the chronic ischemic condition of ACA developed neovascularization so that resistance to ischemia might be increased. Nakanishi et al. had made a similar report, and our patient had likely circulatory insufficiency prior to this incident.[2] In our case, trapping was performed on the right ACA without revascularization. Because there were DAs bilaterally, we could not perform vascular anastomosis.[7] Naturally, trapping the A2 segment without revascularization is associated with risks of ischemia to the ACA territory distal to the trapped vessel. However we suggest that trapping without vascular anastomosis is needed to prevent re-bleeding in certain cases. At such times, it is better to perform a preoperative blood flow study when possible.[4, 7]

## Conclusion

In cases with subarachnoid hemorrhage caused by intracranial dissecting aneurysm, surgical treatment is recommended. But it was impossible to establish vascular anastomosis because the vessel walls of bilateral anterior cerebral artery were very fragile in this case. Trapping without vascular reconstruction was performed at the distal portion of right anterior cerebral artery because of retrograde filling of aneurysmal dissection, and produced a good result. In emergencies trapping without vascular reconstruction may be an option in prevention of rebleeding when good life prognosis is desired.

## Abbreviations

DA: Dissecting aneurysm
CT: Computed tomography
MRI: Magnetic resonance imaging
3D-CT: 3 dimension computed tomography
